# Characterization and antiviral susceptibility of SARS-CoV-2 Omicron/BA.2

**DOI:** 10.21203/rs.3.rs-1375091/v1

**Published:** 2022-02-24

**Authors:** Yoshihiro Kawaoka, Ryuta Uraki, Maki Kiso, Shun Iida, Masaki Imai, Emi Takashita, Makoto Kuroda, Peter Halfmann, Samantha Loeber, Tadashi Maemura, Seiya Yamayoshi, Seiichiro Fujisaki, Zhongde Wang, Mutsumi Ito, Michiko Ujie, Kiyoko Iwatsuki-Horimoto, Yuri Furusawa, Ryan Wright, Zhenlu Chong, Seiya Ozono, Atsuhiro Yasuhara, Hiroshi Ueki, Yuko Sakai, Rong Li, Yanan Liu, Deanna Larson, Michiko Koga, Takeya Tsutsumi, Eisuke Adachi, Makoto Saito, Shinya Yamamoto, Shohei Matsubara, Masao Hagihara, Keiko Mitamura, Tetsuro Sato, Masayuki Hojo, Shin-ichiro Hattori, Kenji Maeda, Moe Okuda, Jurika Murakami, Calvin Duong, Sucheta Godbole, Daniel Douek, Shinji Watanabe, Norio Ohmagari, Hiroshi Yotsuyanagi, Michael Diamond, Hideki Hasegawa, Hiroaki Mitsuya, Tadaki Suzuki

**Affiliations:** University of Wisconsin-Madison; National Center for Global Health and Medicine Research Institute; Institute of Medical Sciences, University of Tokyo; National Institute of Infectious Diseases; University of Tokyo; National Institute of Infectious Diseases; University of Wisconsin at Madison; Influenza Research Institute, Department of Pathobiological Sciences, School of Veterinary Medicine, University of Wisconsin, Madison, WI, USA; University of Wisconsin-Madison; University of Wisconsin; University Of Tokyo; National Institute of Infectious Diseases; Utah State University; University of Tokyo, Institute of Medical Science; Division of Virology, Institute of Medical Science, University of Tokyo; University of Tokyo; Division of Virology, Institute of Medical Science, University of Tokyo; Influenza Research Institute, Department of Pathobiological Sciences, School of Veterinary Medicine, University of Wisconsin-Madison; Washington University; National Institute of Infectious Diseases; Division of Virology, Institute of Medical Science, University of Tokyo; The University of Tokyo; Institute of Medical Sciences, University of Tokyo; Department of Animal Dairy, and Veterinary Sciences, College of Agriculture and Applied Sciences, Utah State University; Department of Animal Dairy, and Veterinary Sciences, College of Agriculture and Applied Sciences, Utah State University; Utah State University, College of Agriculture and Applied Sciences; The Institute of Medical Science, the University of Tokyo; Division of Infectious Diseases, Advanced Clinical Research Center, Institute of Medical Science, University of Tokyo; The Institute of Medical Science, the University of Tokyo; Division of Infectious Diseases, Advanced Clinical Research Center, Institute of Medical Science, University of Tokyo; Division of Infectious Diseases, Advanced Clinical Research Center, Institute of Medical Science, University of Tokyo; Division of Infectious Diseases, Advanced Clinical Research Center, Institute of Medical Science, University of Tokyo; Division of Infection Control, Eiju General Hospital; Division of Infection Control, Eiju General Hospital; Disease Control and Prevention Center, National Center for Global Health and Medicine Hospital; Department of Respiratory Medicine, National Center for Global Health and Medicine Hospital; National Center for Global Health and Medicine Research Institute; National Center For Global Health and Medicine; Department of Virology, Institute of Medical Science, University of Tokyo; Department of Virology, Institute of Medical Science, University of Tokyo; Department of Virology, Institute of Medical Science, University of Tokyo; Human Immunology Section, Vaccine Research Center, National Institute of Allergy and Infectious Diseases, National Institutes of Health; NIH; National Institute of Infectious Diseases; Disease Control and Prevention Center, National Center for Global Health and Medicine; University of Tokyo; Washington University in Saint Louis; National Institute of Infectious Diseases; National Center for Global Health and Medicine Research Institute; National Institute of Infectious Diseases

## Abstract

The recent emergence of SARS-CoV-2 Omicron variants possessing large numbers of mutations has raised concerns of decreased effectiveness of current vaccines, therapeutic monoclonal antibodies, and antiviral drugs for COVID-19 against these variants1,2. While the original Omicron lineage, BA.1, has become dominant in many countries, BA.2 has been detected in at least 67 countries and has become dominant in the Philippines, India, and Denmark. Here, we evaluated the replicative ability and pathogenicity of an authentic infectious BA.2 isolate in immunocompetent and human ACE2 (hACE2)-expressing mice and hamsters. In contrast to recent data with chimeric, recombinant SARS-CoV-2 strains expressing the spike proteins of BA.1 and BA.2 on an ancestral WK-521 backbone3, we observed similar infectivity and pathogenicity in mice and hamsters between BA.2 and BA.1, and less pathogenicity compared to early SARS-CoV-2 strains. We also observed a marked and significant reduction in the neutralizing activity of plasma from COVID-19 convalescent individuals and vaccine recipients against BA.2 compared to ancestral and Delta variant strains. In addition, we found that some therapeutic monoclonal antibodies (REGN10987/REGN10933, COV2-2196/COV2-2130, and S309) and antiviral drugs (molnupiravir, nirmatrelvir, and S-217622) can restrict viral infection in the respiratory organs of hamsters infected with BA.2. These findings suggest that the replication and pathogenicity of BA.2 is comparable to that of BA.1 in rodents and that several therapeutic monoclonal antibodies and antiviral compounds are effective against Omicron/BA.2 variants.

## Introduction

The Omicron (B.1.1.529 lineage) variant of severe acute respiratory syndrome coronavirus 2 (SARS-CoV-2), the virus responsible for coronavirus disease 2019 (COVID-19), was detected in late November 2021 and has spread rapidly around the world. Omicron variants have been classified into four different sublineages: BA.1, BA.1.1, BA.2, and BA.3. The original Omicron lineage, BA.1, has become the dominant variant circulating in many countries; however, BA.2 variants are beginning to dominate in the Philippines, India, and Denmark. Moreover, the prevalence of BA.2 is increasing rapidly in several other countries including South Africa, Sweden, Austria, Singapore, Georgia, and Sri Lanka (https://covariants.org/per-variant). Preliminary data from Denmark and the United Kingdom indicate that the BA.2 variant may be more transmissible than the BA.1 variant^4,5^.

Recently, we and others showed that BA.1 variants are less pathogenic in animal models than previously circulating variants of concern (VOC)^6–8^, consistent with preliminary clinical data in humans^9^. Moreover, other studies have reported that BA.1 variants show reduced sensitivity to vaccine- or infection-induced antibodies, as well as some therapeutic monoclonal antibodies (mAbs)^10–14^. The spike (S) protein of SARS-CoV-2 mediates viral receptor-binding and membrane fusion, both of which are essential for viral infection of host cells. The S protein is also the principal antigen targeted by the host neutralizing antibody response^15^. Importantly, mutations in the S protein, such as E484K N501Y, D614G, and P681H/R, have been shown to affect the infectivity, pathogenicity, transmissibility, species tropism, and/or antigenicity of SARS-CoV-2^16–20^. Compared with the reference strain Wuhan/Hu-1/2019, the BA.1 and BA.2 variants have 36 and 31 amino acid substitutions in the S protein, respectively. Although the *BA.1* and *BA.*2 *variants share* 20 of these substitutions, BA.2 possesses 11 amino acid changes not found in BA.1. These findings suggest that the replicative capacity, pathogenicity, transmissibility, and antigenicity of BA.2 variants may differ from BA.1 variants. Here, we characterized the functional activity of a BA.2 variant *in vivo*. In addition, we evaluated the efficacy of therapeutic mAbs and antiviral drugs for COVID-19 against BA.2 variants *in vivo*.

## Results

### BA.2 and BA.1 Omicron variants show similar attenuated pathogenicity in mice

We isolated a BA.2 variant from a traveler who arrived in Japan from India. The S protein of this isolate contains 31 amino acid changes (Extended Data Table 1) compared to the reference strain Wuhan/Hu-1/2019. These differences include: 7 changes in the N-terminal domain (NTD), including substitutions and deletions (T19L, Δ24-27S, G142D, V213G); 16 substitutions in the Receptor-binding-domain (RBD) (G339D, S371F, S373P, S375F, T376A, D405N, R408S, K417N, N440K, S477N, T478K, E484A, Q493R, Q498R, N501Y, and Y505H); the D614G mutation, three substitutions close to the furin cleavage site (H655Y, N679K, and P681H); and four substitutions in the S2 region (N764K, D796Y, Q954H, and N969K).

**Figure 1:**
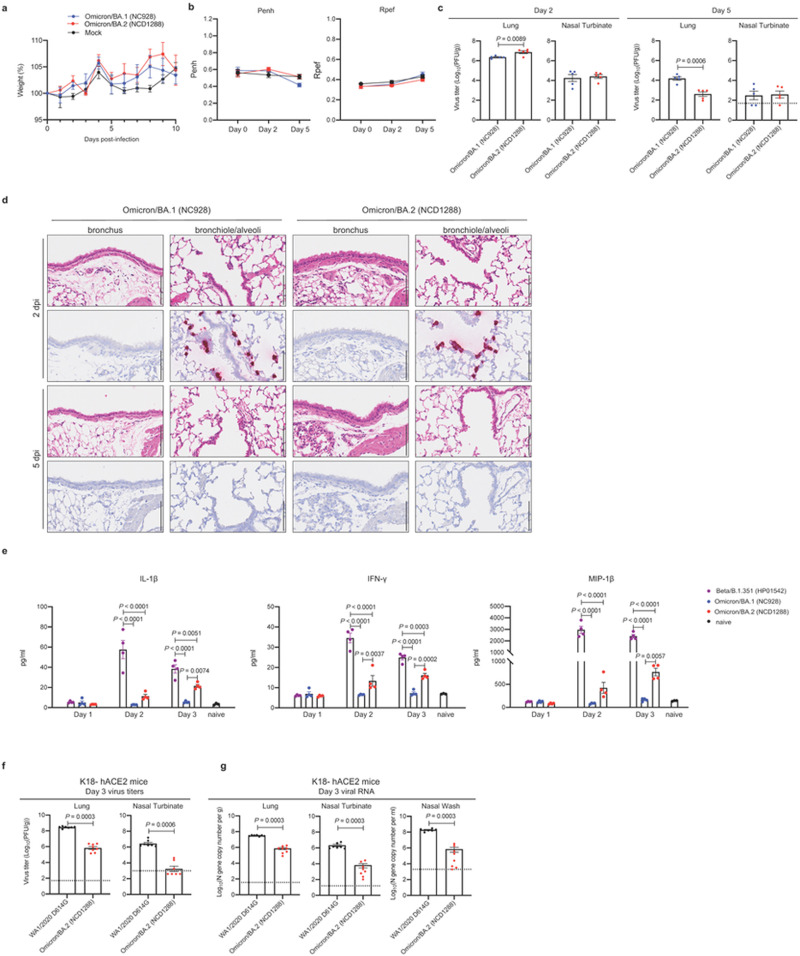
Omicron/BA.2 and Omicron/BA.1 show similar infectivity and pathogenicity in mice. **a–c,** BALB/c mice were intranasally inoculated with 10^5^ PFU in 50 μL of Omicron/BA.1 (NC928), Omicron/BA.2 (NCD1288), or PBS (mock). **a,** Body weight changes in BALB/c mice after viral infection. Body weights of virus-infected (*n* =5) and mock-infected mice (*n* =5) were monitored daily for 10 days. Data are presented as the mean percentages of the starting weight (± s.e.m.). **b,** Pulmonary function analyses in infected mice (*n* =5). Penh and Rpef were measured by using whole-body plethysmography. Mean ± s.e.m. **c,** Virus replication in infected BALB/c mice. Mice (*n* =5) were euthanized at 2 and 5 dpi for virus titration. Virus titers in the nasal turbinates and lungs were determined by plaque assay. Vertical bars show the mean ± s.e.m. Points indicate data from individual mice. The lower limit of detection is indicated by the horizontal dashed line. Data were analyzed by using the Mann-Whitney test. **d,** Histopathological examination of the lungs of infected mice. Three mice per group were infected with 10^5^ PFU of Omicron/BA.1 (NC928) or Omicron/BA.2 (NCD1288) and sacrificed at 2 or 5 dpi for histopathological examinations. Representative images of the bronchi and bronchioles/alveoli of mice infected with BA.1 or BA.2 are shown. Upper panels, hematoxylin and eosin (H&E) staining. Lower panels, *in situ* hybridization targeting the nucleocapsid gene of SARS-CoV-2. Scale bars, 100 μm. **e,** Pro-inflammatory cytokine/chemokine responses in the lungs of infected mice. BALB/c mice were intranasally inoculated with 10^5^ PFU of Beta/B.1.351 (HP01542), BA.1 (NC928), or BA.2 (NCD1288) (infected mice, *n* = 4; naïve mice, *n* =3) at 1, 2, and 3 dpi. Vertical bars show the mean ± s.e.m. Points indicate data from individual mice. Data were analyzed by a two-way ANOVA with Tukey’s multiple comparisons test. **f, g** K18-hACE2 mice were intranasally inoculated with 10^3^ PFU in 50 μL of WA1/2020 D614G or BA.2 (NCD1288). Viral titers (**f**) and RNA levels (**g**) were measured at 3 dpi (*n* = 7–8 per group, 2 experiments). Virus titers in the nasal turbinates and lungs were determined by plaque assay. Mean ± s.e.m. Points indicate data from individual mice. The lower limit of detection is indicated by the horizontal dashed line. Data were analyzed by Mann-Whitney test.

Given that the BA.2 variant possesses substitutions including K417N, E484K, and N501Y in its S protein, and that these amino acids substitutions are key for mouse adaptation^21–23^, we predicted that this variant would infect immunocompetent mice and replicate in their respiratory organs as is seen with BA.1 variants. We therefore inoculated female BALB/c mice with 10^5^ plaque-forming units (PFU) of BA.1 (NC928) or BA.2 (NCD1288), or PBS (mock), and assessed weights for 10 days. Intranasal inoculation of BALB/c mice with BA.1 or BA.2 did not cause body weight changes (Fig. 1a). We also measured pulmonary function in the infected mice by measuring Penh and Rpef, which are surrogate markers for bronchoconstriction and airway obstruction, respectively, by using a whole-body plethysmography (WBP) system. Changes in Penh or Rpef were not observed in the BA.1- or BA.2-infected groups at any timepoint post-infection compared to the mock-infected group (Fig. 1b). In contrast, our previous data showed that B.1.351, which also has the N501Y substitution in its S protein, caused a significant increase in Penh and a decrease in Rpef at 2 days post-infection (dpi)^6^. BALB/c mice infected with BA.1 or BA.2 exhibited similar viral titers in nasal turbinates; however, the mean virus titer of BA.2 in the lungs [mean titer = 6.9 log_10_ (PFU/g)] was slightly but significantly higher than that of BA.1 [mean titer = 6.4 log_10_ (PFU/g)] (Fig. 1c, left panels). At 5 dpi, the lung titers in the BA.2-infected group were lower (33-fold, *P* < 0.001) than those in the BA.1-infected group, although no differences in viral titers in the nasal turbinates were observed between the two groups at this timepoint (Fig. 1c, right panels).

We then performed a histopathological analysis of the lungs of BALB/c mice infected with BA.1 (NC928) or BA.2 (NC1288). In both BA.1- and BA.2-infected mice, inflammatory cell infiltration around the bronchi/bronchioles and in the alveolar spaces was minimal at 2 and 5 dpi (Fig. 1d). *In situ* hybridization revealed that viral RNA was present in the bronchiolar and alveolar epithelium of both BA.1- and BA.2-infected mice, with no differences between the infecting viruses at 2 dpi (Fig. 1d). The distribution of viral antigen, as determined by immunohistochemistry, was similar to that of viral RNA in both BA.1- and BA.2-infected mice (Extended Data Fig. 2a). In both BA.1- and BA.2-infected mice, the amount of detectable viral RNA and antigen decreased over time (Fig. 1d, Extended Data Fig. 2a). These results suggest that while both BA.1 and BA.2 infect bronchiolar and alveolar epithelium in the lungs of BALB/c mice, they displayed substantially less infectivity in the lung of these mice than the Beta variant^6^.

We also assessed the inflammatory responses in the lungs of BALB/c mice. Consistent with our previous study^6^, BA.1 (NC928)-infected BALB/c mice showed similar cytokine/chemokine levels at 1, 2, or 3 dpi as naïve mice. Mice infected with BA.2 (NC1288) had significantly higher levels of several pro-inflammatory cytokines and chemokines, such as IL-1β, IFN-γ, and MIP-1β, compared to mice inoculated with BA.1 at 2 or 3 dpi. However, cytokine and chemokine levels were much lower in the lungs of BA.2 than Beta/B.1.351 (HP01542)-infected mice (Fig. 1e and Extended Data Fig. 1). These results suggest that infection with BA.2 induces a more limited inflammatory response in lungs of mice than B.1.351.

We also evaluated the replication of BA.2 in a more susceptible mouse model^24^, that is, K18-hACE2 transgenic mice, which express hACE2 under the control of an epithelial cytokeratin promoter^25^. At 3 dpi, the virus titers in the lungs and nasal turbinates were substantially (400- to 1000-fold) lower in the respiratory tract of animals infected with BA.2 compared to mice infected with WA1/2020 D614G; the viral RNA levels in the lungs, nasal turbinates, and nasal washes were similarly lower (Fig. 1f, g). Taken together, these findings indicate that Omicron/BA.2 is less pathogenic in mice, similar to that reported for BA.1^6–8^.

### BA.2 and BA.1 Omicron variants show similar replication and pathogenicity in hamsters

**Figure 2:**
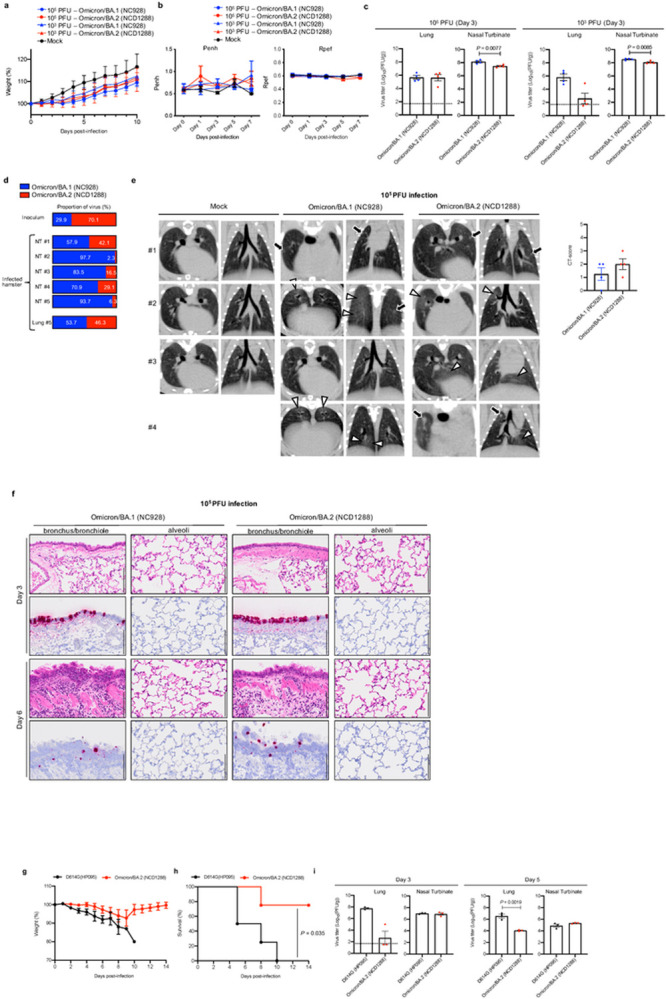
Omicron/BA.2 and Omicron/BA.1 show similar infectivity and pathogenicity in hamsters. **a–c,** Syrian hamsters were intranasally inoculated with 10^5^ or 10^3^ PFU in 30 μL of Omicron/BA.1 (NC928), Omicron/BA.2 (NCD1288), or PBS (mock). **a,** Body weights of virus-infected (*n*=4) and mock-infected hamsters (*n* =3) were monitored daily for 10 days. Data are presented as the mean percentages of the starting weight (± s.e.m.). **b,** Pulmonary function analyses in infected hamsters. Penh and Rpef were measured by using whole-body plethysmography. Mean ± s.e.m. (BA.1- or BA.2-infected hamsters, *n* = 4; mock-infected hamsters, *n* =3). **c,** Virus replication in infected Syrian hamsters. Hamsters (*n* =4) were euthanized at 3 dpi for virus titration. Virus titers in the nasal turbinate and lungs were determined by plaque assay. Vertical bars show the mean ± s.e.m. Points indicate data from individual hamsters. The lower limit of detection is indicated by the horizontal dashed line. Data were analyzed by Mann-Whitney test (titers in the lungs of hamsters infected with 10^3^ PFU) or unpaired student’s t-test (titers in the lungs of hamsters infected with 10^5^ PFU and in the nasal turbinate of hamsters infected with 10^5^ or 10^3^ PFU). **d,** Co-infection with Omicron/BA.1 and Omicron/BA.2. BA.1 (NC928) and BA.2 (NCD1288) were mixed at an equal ratio on the basis of their infectious titers, and the virus mixture (total 2 × 10^3^ PFU) was inoculated into hamsters. Nasal turbinates and lungs were collected from the infected animals at 4 dpi and analyzed using next generation sequencing (NGS). Shown are the relative proportions of BA.1 and BA.2 in the infected animals. **e,** Representative micro-CT axial and coronal images of the lungs of mock-infected hamsters (*n* = 3), and four hamsters per group infected with 10^5^ PFU of Omicron/BA.1 or 10^5^ PFU of Omicron/BA.2 at 7 dpi. Lung abnormalities included minimal, patchy, ill-defined, peri-bronchial ground glass opacity (white arrowheads), and few, small, focal rounded/nodular regions (black arrows), consistent with minimal pneumonia. Coronal CT images were reformatted to optimize lesion visualization. CT severity scores for uninfected hamsters (*n* = 3) or those inoculated with 10^5^ PFU of BA.1 (*n* = 4) or 10^5^ PFU of BA.2 (*n* = 4) were analyzed by using the unpaired student’s t-test. Vertical bars show the mean ± s.e.m. Points indicate data from individual hamsters. **f,** Histopathological examination of the lungs of infected Syrian hamsters. Four hamsters per group were infected with 10^5^ PFU of Omicron/BA.1 (NC928) or Omicron/BA.2 (NCD1288) and sacrificed at 3 or 6 dpi for histopathological examinations. Representative images of the bronchi/bronchioles and alveoli of hamsters infected with BA.1 or BA.2 are shown. Upper panels, hematoxylin and eosin (H&E) staining. Lower panels, *in situ* hybridization targeting the nucleocapsid gene of SARS-CoV-2. Scale bars, 100 μm. **g-i,** hACE2-expressing Syrian hamsters were intranasally inoculated with 10^3^ PFU in 30 μL of D614G (HP095) or Omicron/BA.2 (NCD1288) **g, h,** Body weights (**g**) and survival (**h**) were monitored daily for 14 days. The values for body weights are presented as the mean percentages of the starting weight ± s.e.m. Survival data were analyzed by Log-rank (Mantel-Cox) test. **i**, Three hamsters per group were euthanized at 3 or 5 dpi for virus titration. Virus titers in the nasal turbinates and lungs were determined by use plaque assay. Vertical bars show the mean ± s.e.m. Points indicate data from individual hamsters. The lower limit of detection is indicated by the horizontal dashed line. Data were analyzed by Mann-Whitney test (titers in the lungs at 3 dpi) or unpaired student’s t-test (titers in the lungs at 3 dpi, and those in the nasal turbinates at 3 and 5 dpi). *P* values of < 0.05 were considered statistically significant.

We next evaluated the replication and pathogenicity of the BA.2 variant in Syrian hamsters, a well-established small animal model for the study of COVID-19^26–28^. Syrian hamsters were inoculated with 10^5^ PFU or 10^3^ PFU of BA.1 (NC928) or BA.2 (NCD1288). No differences in weight change were observed between the BA.1- and BA.2-infected groups, with all animals gaining weight (Fig. 2a). Pulmonary function also was evaluated in the infected hamsters using the WBP system. Infection with the BA.2 or BA.1 variant did not cause substantial changes in either Penh or Rpef (Fig. 2b).

Next, we evaluated levels of infection in the respiratory tract of Syrian hamsters. At 3 dpi, in contrast to that seen in mice, virus titers in nasal turbinates were slightly but significantly higher in the hamsters infected with BA.1 than BA. 2, regardless of the inoculation dose (Fig. 2c). Differences in viral titers in the lungs were not observed between animals infected with BA.1 or BA.2 with the high 10^5^ PFU dose at 3 dpi. However, the viral titers in the lungs of hamsters infected with the 10^3^ PFU inoculating dose of BA.2 were lower (>100 fold) than those infected with 10^3^ PFU of BA.1 (Fig. 2c).

To compare the relative fitness and infectivity of BA.1 and BA.2, five hamsters were inoculated with 2 × 10^3^ PFU of a mixture (1:1) of BA.1 (NC928) and BA.2 (NCD1288). At 4 dpi, the nasal turbinates and lungs of the infected hamsters were harvested and assessed by Next Generation Sequencing (NGS) to determine the ratio of BA.1 to BA.2. The ratio was calculated on the basis of the differences between these two viruses across 16 regions in the S protein. NGS analysis revealed that BA.1 became dominant in the nasal turbinates of all five infected animals (Fig. 2d). Due to low read depth, we were unable to determine the ratio in the lung samples from four of the five animals; the sample from the fifth animal showed a slightly greater prevalence of BA.1. These results show that BA.1 outcompetes BA.2 during upper airway tract replication in hamsters.

We next performed microcomputed tomography (micro-CT) to assess for lung abnormalities in hamsters at 7 dpi. We used a previously defined CT severity score (see Legend and Methods) to evaluate animals for ground glass opacities, nodules, and regions of lung consolidation^29^. Micro-CT analysis revealed minimal lung abnormalities in all of the BA.2-infected hamsters, and in 62.5% (5 out of 8) of the BA.1-infected hamsters, including minimal, patchy, ill-defined, peri-bronchial ground glass opacity, and a few, small, focal rounded/nodular regions, consistent with minimal pneumonia (Fig. 2e, Extended Data Fig. 3). These imaging features can be seen with COVID-19 pneumonia, but are nonspecific and can occur with a variety of infectious and noninfectious processes^29^. BA.2-infected hamsters had slightly higher CT severity scores (mean of 1.5 for 10^3^ PFU-inoculated hamsters; mean of 2 for 10^5^ PFU-inoculated hamsters) than BA.1-infected hamsters (mean of 0.75 for 10^3^ PFU-inoculated hamsters; mean of 1.25 for 10^5^ PFU-inoculated hamsters), due to lung abnormalities being present in a slightly higher number of lobes in BA.2-infected hamsters. Thus, the differences in lung abnormalities between BA.2- and BA.1-infected hamsters were subtle based on micro-CT analysis. In contrast to commonly reported imaging features of COVID-19 pneumonia, Syrian hamsters infected with BA.1 or BA.2 showed minimal lung abnormalities and no lung consolidation, which contrasts with earlier studies with ancestral or other VOC^19,26–28,30^, and is consistent with our prior analysis of BA.1 infection^6^.

We also surveyed for histopathological changes in the lungs of BA.1- or BA.2-infected hamsters. This examination revealed that neutrophils and mononuclear cells infiltrate the bronchial/bronchiolar epithelium and subepithelial connective tissues in the bronchi/bronchioles of both BA.1 (NC928)- and BA.2 (NCD1288)-infected hamsters at 6 dpi despite minimal inflammation at 3 dpi (Fig. 2f, Extended Data Fig. 2b). In contrast, there was negligible infiltration of inflammatory cells into the alveolar space of BA.1- and BA.2-infected animals at any timepoint examined (Fig. 2f, Extended Data Fig. 2b). Furthermore, there was no obvious difference in histopathology with different doses of inoculum. Viral RNA-positive or antigen-positive cells were detected in the bronchial epithelium at 3 dpi in both BA.1- and BA.2-infected animals, and more positive cells were seen in the 10^5^ PFU-than the 10^3^ PFU-infected hamsters (Fig. 2f, Extended Data Fig. 2b, 2c). The number of viral RNA-positive or antigen-positive cells decreased over time in both the BA.1- and BA.2-infected animals. There was no obvious difference in the magnitude of inflammation in the bronchi/bronchioles or the distribution of viral RNA and antigen between the BA.1- and BA.2-infected animals. These observations suggest that BA.1 and BA.2 mainly affect bronchi, resulting in bronchitis/bronchiolitis, and that the pathogenicity of Omicron/BA.1 and Omicron/BA.2 is comparable in the hamster model.

We also investigated the infection and pathogenicity of BA.2 using a more susceptible hamster model, that is, transgenic hamsters expressing hACE2 under the control of an epithelial cytokeratin-18 promoter^31^. Intranasal inoculation of hamsters with 10^3^ PFU of D614G (HP095) virus caused remarkable weight loss (> 10%) within the first week (Fig. 2g) and resulted in 100% mortality at 10 dpi (Fig. 2h). In contrast, a small decrease in body weight (< 6%) was observed for most animals infected with BA.2 (NCD1288), similar to our previous findings with BA.1^6^. The lung titers in the BA.2-infected group were approximately 300- to 1,000-fold lower than those in the D614G (HP095)-infected group at 3 and 5 dpi (Fig. 2i), although both viruses replicated to similar levels in the nasal turbinates. These results demonstrate that BA.2 is attenuated in its replication in the lower respiratory tract of hACE2 transgenic hamsters.

Overall, our observations indicate that the replication and pathogenicity in mice and hamsters of BA.2 are similar to those of BA.1 but are attenuated relative to ancestral or other variant strains.

### Antibody responses to the SARS-CoV-2 Omicron/BA.2 variant.

**Figure 3:**
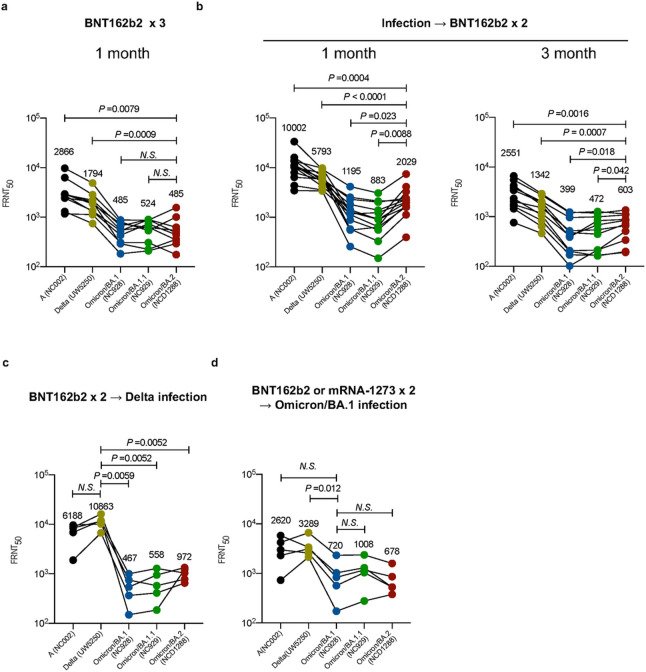
Antibody responses to the SARS-CoV-2 Omicron/BA.2 variant. **a,** Neutralizing antibody titers of human plasma obtained from individuals immunized with a third dose of the BNT162b2 vaccine. Samples were collected 1 month after the third immunization. **b,** Neutralizing antibody titers of human plasma obtained from individuals immunized with two doses of the BNT162b2 vaccine after prior infection. Samples were collected 1 or 3 months after the second immunization. **c,** Neutralizing antibody titers of human plasma obtained from individuals who were infected with the Delta variant after two doses of the BNT162b2 vaccine. Samples were collected 10–95 days after symptom onset. **d,** Neutralizing antibody titers of human plasma obtained from individuals who were infected with the Omicron variant after two doses of the BNT162b2 or mRNA-1273 vaccine. Samples were collected 9–16 days after symptom onset. *P*-values were calculated by one-way ANOVA with a Dunnett’s multiple comparisons test. Each dot represents data from one individual. Geometric mean titers (GMTs) are shown.

Previous studies have established that both BA.1 and BA.1.1 Omicron variants show reduced sensitivity to antibodies in sera and/or plasma from convalescent COVID-19 patients and vaccinated individuals compared to the ancestral strains and other VOC^11,32–36^. We evaluated whether antibodies in plasma from convalescent individuals and vaccine recipients retain neutralizing activity against BA.2. We obtained plasma from four different groups: individuals (1 month post-third dose; *n* = 10) who received three doses of the mRNA vaccine BNT162b2 (Pfizer-BioNTech); individuals [1 (*n =*13) or 3 (*n =*11) month post-second dose] who received two doses of BNT162b2 after prior infection during the first wave; individuals (*n* = 5) who received two doses of BNT162b2 prior to Delta breakthrough infection; and individuals (*n* = 5) who received two doses of BNT162b2 or the mRNA-1273 (Moderna) vaccine prior to Omicron breakthrough infection. Neutralization titers against BA.2 were determined by using authentic BA.2 and a focus reduction neutralization test (FRNT), and compared with those against an ancestral SARS-CoV-2 strain (SARS-CoV-2/UT-NC002-1T/Human/2020/Tokyo; NC002) from February 2020, Delta (UW-5250), BA.1, and BA.1.1 (Fig. 3a and 3b).

The 50% FRNT (FRNT_50_) geometric mean titers of plasma from individuals immunized with a third dose of the BNT162b2 vaccine against BA.1, BA1.1, and BA.2 were significantly reduced (by 3.4- to 5.9-fold) compared with those against the ancestral and Delta strains (Fig. 3a). This reduction in neutralizing titers was similar between the three Omicron viruses. FRNT_50_ geometric mean titers against BA.1, BA1.1, and BA.2 after administration of a second dose of the BNT162b2 vaccine in previously infected individuals were 2.9- to 6.5-fold lower than those against the ancestral and Delta strains; however, this reduction in neutralizing titers was larger for BA.1 and BA.1.1 than for BA.2 at both 1- and 3-months post-vaccination (Fig. 3b). All of the plasma samples from the COVID-19 vaccine recipients who experiencing Delta breakthrough infections showed high FRNT_50_ values against the Delta variant (Fig. 3c). However, this breakthrough infection induced low levels of neutralizing antibodies against BA.1, BA.1.1, and BA. 2 compared to the Delta variant. A similar trend was observed for samples from vaccine recipients who experienced Omicron breakthrough infection (Fig. 3d). Despite the fact that these individuals were infected with Omicron variants, the FRNT_50_ geometric mean titers of their plasma samples against the three Omicron strains still were 3.3- to 4.9-fold lower than against the Delta variant. Collectively, these results suggest that antibodies elicited by the COVID-19 mRNA vaccine and/or SARS-COV-2 infection have reduced neutralizing activity against BA.2 compared to other Omicron strains.

### Therapeutic effects of monoclonal antibodies (mAbs) directed against the BA.2 variant.

**Figure 4:**
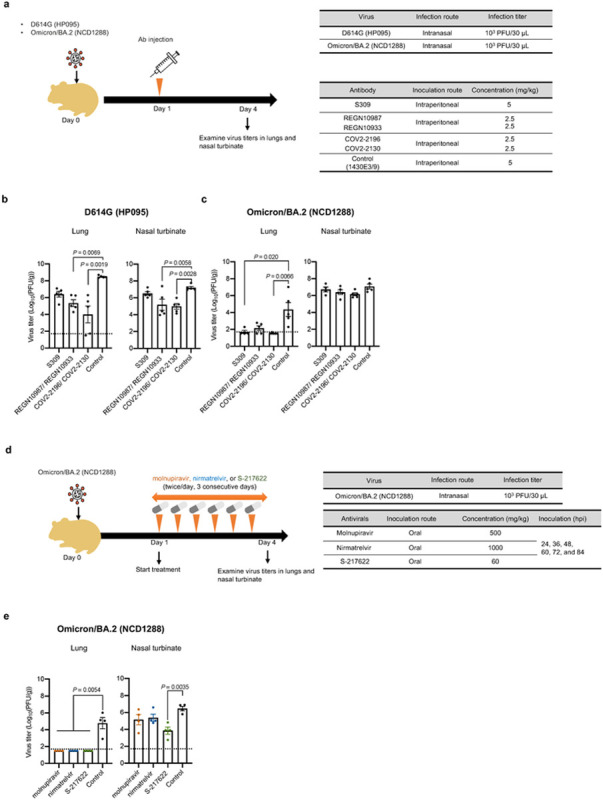
Therapeutic effects of monoclonal antibodies and antiviral compounds against SARS-CoV-2 Omicron variants. **a,** Diagram of the experimental workflow for assessing the therapeutic effects of monoclonal antibodies. **b,** Syrian hamsters were intranasally inoculated with 10^3^ PFU of Omicron/BA.2 (NCD1288) or D614G (HP095). One day after infection, hamsters were intraperitoneally injected with a single dose of the REGN10987/REGN10933 or COV2-2196/COV2-2130 combination (2.5 mg/kg each), or S309 as monotherapy (5 mg/kg). A human monoclonal antibody (1430E3/9) against the hemagglutinin of influenza B virus was injected as a control. Four to five hamsters per group were euthanized at 4 dpi for virus titration. **c** Diagram of the experimental workflow for assessing the therapeutic effects of antiviral compounds. **d** Syrian hamsters were intranasally inoculated with 10^3^ PFU of Omicron/BA.2 (NCD1288). One day after infection, hamsters were treated with: 500 mg/kg molnupiravir, 1000 mg/kg nirmatrelvir, or 60 mg/kg S-217622 orally twice daily for 3 days. Methylcellulose served as a control for oral treatment. Four hamsters per group were euthanized at 4 dpi for virus titration. Virus titers in the nasal turbinates and lungs were determined by plaque assay. Vertical bars show the mean ± s.e.m. Points indicate data from individual hamsters. The lower limit of detection is indicated by the horizontal dashed line. To compare the lung and nasal turbinate titers of the Omicron/BA.2 (NCD1288)- and D614G (HP095)-infected hamster groups, we used a Kruskal-Wallis test with Dunn’s multiple comparisons test and a one-way ANOVA with Dunnett’s multiple comparisons test, respectively. *P* values of < 0.05 were considered statistically significant.

We recently evaluated the therapeutic effect of FDA-approved mAbs against a BA.1 variant in hamsters^37^. Our data suggest that certain mAbs may lose efficacy against this variant. We, therefore, asked whether therapeutic mAbs can inhibit infection of BA.2 variants in hamsters. As of February, 2022, the FDA has authorized the emergency use of four mAbs for the treatment and/or prevention of COVID-19: *REGEN-COV*, a combination of imdevimab (REGN10987) and casirivimab (REGN10933); Xevudy, which is sotrovimab (VIR-7831); Evusheld (AZD7442), a combination of tixagevimab (COV2-2196 or AZD8955) and cilgavimab (COV2-2130 or AZD1061); and bebtelovimab (LY-CoV1404). We tested REGN10987/REGN10933, S309 (the precursor of sotrovimab), and COV2-2196/COV2-2130 for their therapeutic efficacy against BA.2. We synthesized these mAbs according to publicly available sequences without modifications in their Fc regions, as described previously^14,37^. Syrian hamsters were inoculated with 10^3^ PFU of CoV-2/UT-HP095-1N/Human/2020/Tokyo (D614G; HP095) or BA.2 (NCD1288), and one day later were injected intraperitoneally with S309 (5 mg/kg), the REGN10987/REGN10933 combination (2.5 mg/kg each), or the COV2-2196/COV2-2130 combination (2.5 mg/kg each) (Fig. 4a). A mAb specific to the hemagglutinin of influenza B virus was used as a control. Sera were also collected at this timepoint and tested in an ELISA for RBD-specific IgG antibodies, which confirmed successful antibody transfer. Similar to our previous experiment^37^, for the D614G (HP095)-infected groups, treatment with S309, REGN10987/REGN10933, or COV2-2196/COV2-2130 markedly reduced virus titers in the lungs compared to those treated with the control mAb (Fig. 4b). For the BA.2 (NCD1288)-infected groups, COV2-2196/COV2-2130 treatment suppressed virus infection in the lungs of the animals [mean reduction in viral titer = 2.9 log_10_ (PFU/g)] (Fig. 4c), similar to our previous findings with BA.1 (NC928)^37^. In addition, S309 and REGN10987/REGN10933, which we previously showed were not effective against BA.1, reduced lung virus titers [mean reduction in viral titer = 2.7 and 2.2 log_10_ (PFU/g), respectively], although the difference was not statistically significant between the REGN10987/REGN10933- and control mAb-treated groups. A recent study showed that the neutralizing activity of S309 against BA.2 is lower than that against BA.1^38^. The disparity between the *in vitro* and *in vivo* findings may be due in part to the attenuated infectivity (~100-fold lower levels) of BA.2 compared to BA.1 in hamster lungs (Fig. 2c). None of the mAbs we tested affected the virus titers in the nasal turbinates of the animals. These results suggest that REGN10987/REGN10933, COV2-2196/COV2-2130, and S309 can restrict viral replication in the lungs of hamsters infected with BA.2 when these mAbs are administrated therapeutically.

### Effects of antiviral compounds on the replication of SARS-CoV-2 Omicron/BA.2 variants.

Molnupiravir, an inhibitor of the RNA-dependent RNA polymerase of SARS-CoV-2, and nirmatrelvir^39^, an inhibitor of the main protease (also called 3CLpro) of SARS-CoV-2, have been authorized for emergency use by the FDA to treat COVID-19. In addition, S-217622, another inhibitor of 3CLpro, is currently in clinical trials^37,40^. We assessed the therapeutic efficacy of these compounds in hamsters infected with BA.2 (NCD1288). Hamsters intranasally inoculated with 10^3^ PFU of BA.2 (NCD1288) were treated via oral gavage twice daily (at 12-h intervals) for 1, 2, or 3 days with 500 mg/kg/12 h (1000 mg/kg/day) of molnupiravir, 60 mg/kg/12 h (120 mg/kg/day) of S-217622, or 1000 mg/kg/12 h (2000 mg/kg/day) of nirmatrelvir, beginning 24 h post-infection (Fig. 4d). These dosages were selected based on previous studies in mice or hamsters^37,41,42^. Although no differences in viral titers in the nasal turbinates were detected between the animals treated with molnupiravir or nirmatrelvir and those treated with methylcellulose (control) at 4 dpi, treatment with S-217622 significantly reduced the virus titers in the nasal turbinates at 4 dpi. Moreover, all of the compounds tested considerably reduced the lung virus titers; no infectious virus was recovered at 4 dpi from the lungs of animals treated with molnupiravir, nirmatrelvir, or S-217622 (Fig. 4e). Our data suggest that the three antiviral compounds tested effectively inhibit BA.2 replication in the lower respiratory tract of hamsters. In addition, animals treated with S-217622 showed reduced infection in the upper respiratory tract, consistent with a previous study^37^.

## Discussion

Here we found that the Omicron sublineage BA.2 variant of SARS-CoV-2 is less pathogenic in rodent models than prior SARS-CoV-2 variants, as has been reported for Omicron sublineage BA.1 variants^6–8^. We observed that a BA.2 variant was limited in its infectivity in the lungs of mice and hamsters compared to previous variants. Moreover, infection with BA.2 induced a limited pro-inflammatory cytokine/chemokine response in mouse lungs. Importantly, histopathological examination revealed that viral RNA and antigen were rarely detected in the alveoli of mice and hamsters after infection with BA.2. Thus, the limited infectivity of the BA.2 variant in the lung might contribute to lower disease severity in animal models. Further investigation is required to determine whether BA.2 variants can replicate efficiently and equivalently in human alveolar epithelial cells.

Our study suggests that the pathogenicity and replicative ability of the BA.2 variant is comparable to that of BA.1 variants in animal models. In contrast, a recent study showed that a recombinant, chimeric SARS-CoV-2 possessing the BA.2 S gene in the background of an ancestral SARS-CoV-2 strain is more pathogenic than a recombinant virus bearing the BA.1 S gene in the same background^3^, suggesting that the differences in the BA.1 and BA.2 S genes are responsible for the difference in pathogenicity between these chimeric viruses; based on these data, the authors concluded that BA.2 is more pathogenic than BA.1. However, in natural isolates, the genomes of BA.1 and BA2 also differ at locations other than the S gene, and these differences could have epistatic or independent effects that offset differences in pathogenic potential of the S gene^43^.

The *RBD* of the S protein is the primary target for therapeutic antibodies and antibodies elicited by vaccination or infection. BA.2 has 16 amino acid substitutions in its RBD compared to the reference strain Wuhan/Hu-1/2019. *BA.2* and *BA.1 variants share* 12 of these 16 substitutions; BA.2 possesses four RBD substitutions (i.e., S371F, T376A, D405N, and R408S) that differ from those in BA.1, which *could affect the* efficacy of therapeutic mAbs and mRNA vaccines against the different Omicron sublineages. We found that S309, REGN10987/REGN10933, and COV2-2196/COV2-2130 inhibited BA.2 replication in the lungs of hamsters. In contrast, our previous study showed that the therapeutic administration of S309 or REGN10987/REGN10933 had no effect on the virus titers in the lungs of hamsters infected with BA.1^37^. These findings highlight that, when treating COVID-19 patients with mAb-based therapy, the prevailing Omicron sublineage should be considered.

A marked and significant reduction in neutralizing activity was observed against BA.2 compared to the ancestral and Delta strains for convalescent individuals and vaccinees. Interestingly, plasma from individuals infected with early pandemic SARS-CoV-2 strains and then vaccinated with the BNT162b2 vaccine showed higher neutralizing activity against BA.2 than against BA.1 and BA.1.1 (Fig. 3). A recent study using *in vitro* neutralization assays reported that *there were differences in the* reactivity of several mAbs against these different Omicron sublineages^38^. These observations suggest that BA.2 are antigenically distinct from BA.1 and BA.1.1, which is consistent with the data performed convalescent hamster sera^3^.

We note several limitations in this study: (1) We characterized only one BA.2 isolate. It is possible that the other BA.2 strain(s) may have substitution(s) that are not present in the isolate we tested, which could affect infectivity and/or pathogenicity of SARS-CoV-2; (2) The relationships between neutralizing titers and protection against COVID-19 in humans remains unclear. Future clinical studies should assess how much neutralizing activity is required to prevent Omicron variant infections and predict vaccine efficacy; and (3) Although hamsters are one of the most susceptible animals to SARS-CoV-2 among those tested, including mice and non-human primates, the Omicron/BA.2 variant is attenuated in the lungs of hamsters. It is unclear whether the BA.2 variant in humans is attenuated to the degree that it is in hamsters. Differences in replication of Omicron variants in humans and hamsters could affect the effectiveness of the mAbs and antiviral compounds. Clinical data on the efficacy of the mAbs and antiviral drugs for the treatment of patients infected with BA.2 are needed to corroborate the findings in the hamster model.

In summary, our data suggest that the Omicron/BA.2 variant of SARS-CoV-2 has similar infectivity and pathogenicity as the Omicron/BA.1 variant in rodent models and is susceptible to restriction by several therapeutic monoclonal antibodies and antiviral compounds in clinical use or development.

## Materials And Methods

### Cells.

VeroE6/TMPRSS2 (JCRB 1819) cells^30,44^ were propagated in the presence of 1 mg/ml geneticin (G418; Invivogen) and 5 μg/ml plasmocin prophylactic (Invivogen) in Dulbecco’s modified Eagle’s medium (DMEM) containing 10% Fetal Calf Serum (FCS). Vero-hACE2-TMPRSS2 cells were cultured in DMEM supplemented with 10% FCS, 10 mM HEPES pH 7.3, and 100 U/mL of penicillin–streptomycin 10 μg/mL of puromycin. VeroE6/TMPRSS2 and Vero-hACE2-TMPRSS2 cells were maintained at 37 °C with 5% CO_2_.

Chinese hamster ovary (CHO) cells were maintained in DMEM containing 10% FCS and antibiotics at 37 °C with 5% CO_2_. Expi293F cells (Thermo Fisher Scientific) were maintained in Expi293 expression medium (Thermo Fisher Scientific) at 37 °C under 8% CO_2_. The cells were regularly tested for mycoplasma contamination by using PCR, and confirmed to be mycoplasma-free.

### Clinical specimens.

After informed consent was obtained, respiratory specimens were collected from SARS-CoV-2-infected individuals and plasma specimens were collected from COVID-19 convalescent individuals and vaccinees. The research protocol was approved by the Research Ethics Review Committee of the Institute of Medical Science of the University of Tokyo (approval number: 2019–71–0201 and 2020-74-0226).

### Viruses.

hCoV-19/Japan/UT-NCD1288-2N/2022 (Omicron/BA.2; NCD1288), hCoV-19/Japan/NC928-2N/2021 (Omicron/BA.1; NC928)^14^, hCoV-19/Japan/NC929-1N/2021 (Omicron/BA.1.1; NC929)^37^, SARS-CoV-2/UT-HP095-1N/Human/2020/Tokyo (D614G; HP095)^30^, hCoV-19/USA/MD-HP01542/2021 (Beta; HP01542), hCoV-19/USA/WI-UW-5250/2021 (Delta; UW5250), and SARS-CoV-2/UT-NC002-1T/Human/2020/Tokyo (NC002) were propagated in VeroE6/TMPRSS2 cells in VP-SFM (Thermo Fisher Scientific). The SARS-CoV-2/USA-WA1/2020 (WA1/2020) recombinant strain with substitution (D614G) was described previously^45^. Omicron/BA.2 (NCD1288) was subjected to NGS as described^46^; sequence differences between Omicron/BA.2 (NCD1288) and the reference sequence (Wuhan/Hu-1/2019) are shown in Extended Data Table 1.

All experiments with SARS-CoV-2 were performed in enhanced biosafety level 3 (BSL3) containment laboratories at the University of Tokyo and the National Institute of Infectious Diseases, Japan, which are approved for such use by the Ministry of Agriculture, Forestry, and Fisheries, Japan. Animal studies at Washington University were performed in approved A-BSL3 facilities using appropriate personal protection and positive pressure respirators.

### Antibodies.

Amino acid sequences for the variable region of the heavy and light chains of the following human monoclonal antibodies against the S protein were used for gene synthesis: clones tixagevimab (COV2-2196/AZD8895; GenBank accession numbers QLI33947 and QLI33948), casirivimab (REGN10933; PDB accession numbers 6XDG_B and 6XDG_D), cilgavimab (COV2-2130/AZD1061; GenBank accession numbers QKY76296 and QKY75909), imdevimab (REGN10987; PDB accession numbers 6XDG_A and 6XDG_A), and S309 (PDB accession numbers 6WS6_A and 6WS6_F). An artificial signal sequence and the constant gamma heavy (IgG1, UniProtKB/Swiss-Prot accession number P01857) and kappa (UniProtKB/Swiss-Prot accession number P01834) or lambda (UniProtKB/Swiss-Prot accession number P0DOY2) light chain coding sequences were added before and after each variable region. Codon usage was optimized for expression in CHO cells. The synthesized genes were cloned into a plasmid for protein expression and transfected into CHO cells. Cell culture media were harvested after incubation for 10–14 days at 37°C. A human monoclonal antibody (1430E3/9) against the hemagglutinin of influenza B virus was previously cloned into the expression vector Mammalian Power Express System (TOYOBO)^47^ and was transiently expressed by Expi293F cells. Monoclonal antibodies were purified by using MabSelect SuRe LX (Cytiva) or a protein A column. Purity was confirmed by SDS-PAGE and/or HPLC before use. The reactivities of these antibodies against SARS-CoV-2, including the Alpha, Beta, Delta, Gamma, and Omicron variants, have been tested previously.

### Antiviral compounds.

Molnupiravir (EIDD-2801) and nirmatrelvir (PF-07321332) were purchased from MedChemExpress. S-217622 was kindly provided by Shionogi Co., Ltd. All compounds were dissolved in 0.5% methylcellulose prior to use in *in vivo* experiments.

### Animal experiments and approvals.

Animal studies were carried out in accordance with the recommendations in the Guide for the Care and Use of Laboratory Animals of the National Institutes of Health. The protocols were approved by the Institutional Animal Care and Use Committee at the Washington University School of Medicine (assurance number A3381-01), University of Wisconsin, Madison (V006426) and the Animal Experiment Committee of the Institute of Medical Science, the University of Tokyo (approval numbers PA19-72 and PA19-75). Virus inoculations were performed under anesthesia, and all efforts were made to minimize animal suffering. *In vivo* studies were not blinded, and animals were randomly assigned to infection groups. No sample-size calculations were performed to power each study. Instead, sample sizes were determined based on prior *in vivo* virus challenge experiments.

### Experimental infection of Syrian hamsters.

Five- to six-week-old male wild type Syrian hamsters (Japan SLC Inc., Shizuoka, Japan) were used in this study. Baseline body weights were measured before infection. Under *isoflurane* anesthesia, four hamsters per group were intranasally inoculated with 10^3^ PFU (in 30 μL) or with 10^5^ PFU (in 30 μL) of Omicron/BA.2 (NCD1288) or Omicron/BA.1 (NC928). Body weight was monitored daily for 10 days. For virological and pathological examinations, four hamsters per group were intranasally infected with 10^3^ PFU or 10^5^ PFU of virus; 3 and 6 dpi, the animals were euthanized and nasal turbinates and lungs were collected. The virus titers in the nasal turbinates and lungs were determined by use of plaque assays on VeroE6/TMPRSS2 cells.

For co-infection studies, BA.1 (NC928) and BA.2 (NCD1288) were mixed at an equal ratio on the basis of their titers, and the virus mixture (total 2 × 10^3^ PFU) was inoculated into hamsters. The animals were euthanized and nasal turbinates and lungs were collected at 4 dpi.

The K18-hACE2 transgenic hamster line (line M51; the same line as our previous study^6^) was developed with a piggyBac-mediated transgenic approach, in which the K18-hACE2 cassette from the pK18-hACE2 plasmid was transferred into a piggyBac vector, pmhyGENIE-356, for pronuclear injection. hACE2 transgenic hamsters will be described in detail elsewhere^31^. Male K18-hACE2 transgenic hamsters were intranasally inoculated with 10^3^ PFU of D614G (HP-095) or Omicron/BA.2 (NCD1288) in 30 μL. Body weight and survival were monitored daily, and nasal turbinates and lungs were collected at 3 and 5 dpi for virological analysis.

### Experimental infection of mice.

Heterozygous K18-hACE2 C57BL/6J mice (strain 2B6.Cg-Tg(K18-ACE2)2Prlmn/J) were obtained from the Jackson Laboratory. BALB/c mice were purchased from Japan SLC Inc.

Twelve-week-old female K18-hACE2 mice were inoculated via the intranasal route with 10^5^ PFU (in 50 μL) of BA.2 (NCD1288) or WA1/2020 D614G; At 3 dpi, the animals were euthanized and lungs were collected. The virus titers in the nasal turbinates and lungs were determined by plaque assays on VeroE6/TMPRSS2-hACE cells. To measure viral RNA levels, nasal washes were collected in 0.5 mL of PBS, and tissues were weighed and homogenized with zirconia beads in a MagNA Lyser instrument (Roche Life Science) in 1 mL of DMEM supplemented with 2% FBS. Tissue homogenates were clarified by centrifugation at 10,000 rpm for 5 min and stored at −80 °C. Viral RNA from homogenized tissues or nasal washes was isolated by using the MagMAX Viral RNA Isolation Kit (ThermoFisher) and measured by TaqMan one-step quantitative reverse-transcription PCR (RT-qPCR) on an ABI 7500 Fast Instrument. Copies of SARS-CoV-2 N gene RNA in samples were determined by using a previously published assay^48^. Briefly, a TaqMan assay was designed to target a highly conserved region of the N gene (Forward primer: ATGCTGCAATCGTGCTACAA; Reverse primer: GACTGCCGCCTCTGCTC; Probe: /56-FAM/TCAAGGAAC/ZEN/AACATTGCCAA/3IABkFQ/).

Five-week-old female BALB/c mice were intranasally inoculated with 10^5^ PFU (in 50 μL) of BA.1 (NC928) or BA.2 (NCD1288) under *isoflurane* anesthesia. Body weights were measured before infection and then daily. Two and 5 dpi, the animals were euthanized and nasal turbinates and lungs were collected. The virus titers in the nasal turbinates and lungs were determined by plaque assays on VeroE6/TMPRSS2 cells.

### Lung function.

Respiratory parameters were measured by using a whole-body plethysmography system (PrimeBioscience) according to the manufacturer’s instructions. In brief, hamsters were placed in the unrestrained plethysmography chambers and allowed to acclimatize for 1 min before data were acquired over a 3-min period by using FinePointe software. Mice were placed in the unrestrained plethysmography chambers and allowed to acclimatize for 5 min before data were acquired over a 5-min period by using FinePointe software.

### Micro-CT imaging.

Hamsters were inoculated intranasally with 10^3^ PFU (in 30 μL) or 10^5^ PFU (in 30 μL) of BA.1 (NC928), BA.2 (NCD1288), or PBS. Lungs of infected animals were imaged by using an in *vivo* micro-CT scanner (CosmoScan FX; Rigaku Corporation, Japan). Under ketamine-xylazine anesthesia, the animals were placed in the image chamber and scanned for 2 min at 90 kV, 88 μA, FOV 45 mm, and pixel size 90.0 μm. After scanning, the lung images were reconstructed by using the CosmoScan Database software of the micro-CT (Rigaku Corporation, Japan) and analyzed by using the manufacturer-supplied software.

A CT severity score, adapted from a human scoring system, was used to grade the severity of the lung abnormalities^49^. Each lung lobe was analyzed for degree of involvement and scored from 0–4 depending on the severity: 0 (none, 0%), 1 (minimal, 1%–25%), 2 (mild, 26%–50%), 3 (moderate, 51%–75%), or 4 (severe, 76%–100%). Scores for the five lung lobes were summed to obtain a total severity score of 0–20, reflecting the severity of abnormalities across the five groups. Images were anonymized and randomized; the scorer was blinded to the group allocation.

### Pathology.

Excised animal tissues were fixed in 4% paraformaldehyde in PBS, and processed for paraffin embedding. The paraffin blocks were cut into 3-μm-thick sections and mounted on silane-coated glass slides for histopathological examination. To detect SARS-CoV-2 RNA, *in situ* hybridization was performed using an RNA scope 2.5 HD Red Detection kit (Advanced Cell Diagnostics, Newark, California) with an antisense probe targeting the nucleocapsid gene of SARS-CoV-2 (Advanced Cell Diagnostics) as previously described^6^. Tissue sections were also processed for immunohistochemical staining with a rabbit polyclonal antibody for SARS-CoV nucleocapsid protein (ProSpec; ANT-180, Rehovot, Israel), which cross-reacts with SARS-CoV-2 nucleocapsid protein. Specific antigen-antibody reactions were visualized by means of 3,3’-diaminobenzidine tetrahydrochloride staining using the Dako Envision system (Dako Cytomation, Glostrup, Denmark).

### Analysis of the ratio of BA.1 and BA.2 viruses after co-infection.

Viral RNA was extracted by using a QIAamp Viral RNA Mini Kit (QIAGEN). The whole genome of SARS-CoV-2 was amplified by using a modified ARTIC network protocol in which some primers were replaced or added^50,51^. Briefly, viral cDNA was synthesized from the extracted RNA by using a LunarScript RT SuperMix Kit (New England BioLabs). The DNA was amplified by performing a multiplexed PCR in two pools using the ARTIC-N1 primers v5^52^ and the Q5 Hot Start DNA polymerase (New England BioLabs). The DNA libraries for Illumina NGS were prepared from pooled amplicons by using a QIAseq FX DNA Library Kit (QIAGEN) and were then analyzed by using the iSeq 100 System (Illumina). The reads were assembled by the CLC Genomics Workbench (version 21, Qiagen) with the Wuhan/Hu-1/2019 sequence (GenBank accession no. MN908947) as a reference. The ratio of BA.1 and BA.2 was calculated from the differences between these two viruses across 16 regions. The nucleotide numbers in these 16 regions are: 21618, 21633–21641, 21762, 21765–21770, 21846, 21987–21995, 22194–22196, 22200, 22688, 22775, 22786, 22898, 23048, 23202, 24130, and 24503 in the SARS-CoV-2 genome, which correspond to amino acids 19, 25– 27, 67, 69–70, 95, 143–145, 211–212, 213, 376, 405, 408, 446, 496, 547, 856, and 981 in the spike protein, respectively. Samples with more than 100 read-depths were analyzed.

### Evaluation of therapeutic efficacy of mAbs and antiviral compounds in Syrian hamsters.

Five- to six-week-old male Syrian hamsters (Japan SLC Inc., Shizuoka, Japan) were used in this study. For the evaluation of mAb efficacy in hamsters, under *isoflurane* anesthesia, five hamsters per group were inoculated intranasally with 10^3^ PFU (in 30 μL) of BA.2 (NCD1288) or D614G (HP095). Twenty-four hours after infection, the hamsters were injected intraperitoneally with 1 ml of a mAb preparation (5 mg/kg). The animals were euthanized at 4 dpi, and the virus titers in the nasal turbinates and lungs were determined by plaque assays on VeroE6/TMPRSS2 cells.

For the evaluation of antiviral compound efficacy in hamsters, under *isoflurane* anesthesia, four hamsters per group were inoculated intranasally with 10^3^ PFU (in 30 μL) of BA.2 (NCD1288). At 24 h after inoculation, hamsters were treated with the following antiviral compounds: (1) molnupiravir, 500 mg/kg (in 1 ml) administered orally twice daily; (2) nirmatrelvir, 1,000 mg/kg (in 1 ml) administered orally twice daily; (3) S-217622, 60 mg/kg (in 1 ml) administered orally twice daily; or (4) methylcellulose (1 ml) as a control for oral treatment. The animals were euthanized at 4 dpi, and the virus titers in the nasal turbinates and lungs were determined by plaque assays on VeroE6/TMPRSS2 cells.

### Cytokine and chemokine measurement

Under *isoflurane* anesthesia, five-week-old female BALB/c mice were intranasally inoculated with 10^5^ PFU (in 50 μL) of Beta (HP01542), BA.1 (NC928) or BA.2 (NCD1288); 1, 2, and 3 dpi, the animals were euthanized, and lungs were collected. For cytokine and chemokine measurement, homogenates of mouse lungs were processed with the Bio-Plex Mouse Cytokine 23-Plex and 9-Plex panels (Bio-Rad Laboratories).

### Enzyme-Linked Immunosorbent Assay (ELISA).

ELISAs were performed as previously reported^53^. Briefly, 96-well Maxisorp microplates (Nunc) were incubated with the recombinant receptor-binding domain (RBD) of the S protein or HexaPro prefusion-stabilized versions of the S ectodomain (S_6pro_) (prototype virus or omicron variant) (50 μL/well at 2 μg/ml), or with PBS at 4 °C overnight and were then incubated with 5% skim milk in PBS containing 0.05% Tween-20 (PBS-T) for 1 h at room temperature. The microplates were reacted for 1 h at room temperature with hamster serum samples that has been diluted 40-fold or with 1 μg/ml monoclonal antibody in PBS-T containing 5% skim milk and subsequently serially 2-fold diluted, followed by peroxidase-conjugated goat anti-human IgG, Fcγ Fragment specific antibody (Jackson Immuno-Research) for 1 h at room temperature. Then, 1-Step Ultra TMB-Blotting Solution (Thermo Fisher scientific) was added to each well and incubated for 3 min at room temperature. The reaction was stopped by the addition of 2 M H_2_SO_4_ and the optical density at 450 nm (OD_450_) was immediately measured. The average OD_450_ values of two PBS-wells were subtracted from the average OD_450_ values of the two RBD or S_6pro_-wells for background correction. A subtracted OD_450_ value of 0.1 or more was regarded as positive; the minimum dilution to give a positive result was used as the ELISA titer (hamster serum) or minimum concentration to bind the S protein (monoclonal antibodies).

### Focus reduction neutralization test (FRNT)

Neutralization activities of SARS-CoV-2 were determined by using an FRNT as previously described^14^. Serial dilutions of plasma were mixed with 1,000 focus-forming units of virus/well and incubated for 1 h at 37 °C. The antibody-virus mixture was inoculated on VeroE6/TMPRSS2 cells in 96-well plates in duplicate and incubated for 1 h at 37 °C. An equal volume of 1.2% Avicel RC-581 (DuPont Nutrition USA) in culture medium was added to each well. The cells were incubated for 24 h at 37 °C and then fixed with formalin. After the formalin was removed, the cells were immunostained with a mouse monoclonal antibody against SARS-CoV-1/2 nucleoprotein [clone 1C7C7 (Sigma-Aldrich)], followed by a horseradish peroxidase-labeled goat anti-mouse immunoglobulin (SeraCare Life Sciences). The infected cells were stained with TrueBlue Substrate (SeraCare Life Sciences) and then washed with distilled water. After cell drying, the focus numbers were quantified by using an ImmunoSpot S6 Analyzer, ImmunoCapture software, and BioSpot software (Cellular Technology). The results are expressed as the 50% focus reduction neutralization titer (FRNT_50_). The FRNT_50_ values were calculated by using GraphPad Prism (GraphPad Software).

### Statistical analysis.

GraphPad Prism software was used to analyze all of the data. Statistical analysis included unpaired t tests, Mann-Whitney tests, Log-rank (Mantel-Cox) test and ANOVA with multiple corrections post-test. Differences among groups were considered significant for *P* values < 0.05.

### Data availability.

All data supporting the findings of this study are available within the paper and from the corresponding author upon request. There are no restrictions to obtaining access to the primary data.

### Code availability.

No code was used in the course of the data acquisition or analysis.

### Reagent availability.

All reagents described in this paper are available through Material Transfer Agreements.

## Supplementary Material

Supplement 1

Supplement 2

Supplement 3
