# Supplementary figures and images for: Characterization and antiviral susceptibility of SARS-CoV-2 Omicron/BA.2
Source: Res Sq. 2022 Feb 24:rs.3.rs-1375091. Preprint. [Version 1] doi: 10.21203/rs.3.rs-1375091/v1 (PMC8887076; doi:10.21203/rs.3.rs-1375091/v1)

Extended Data Figure 1

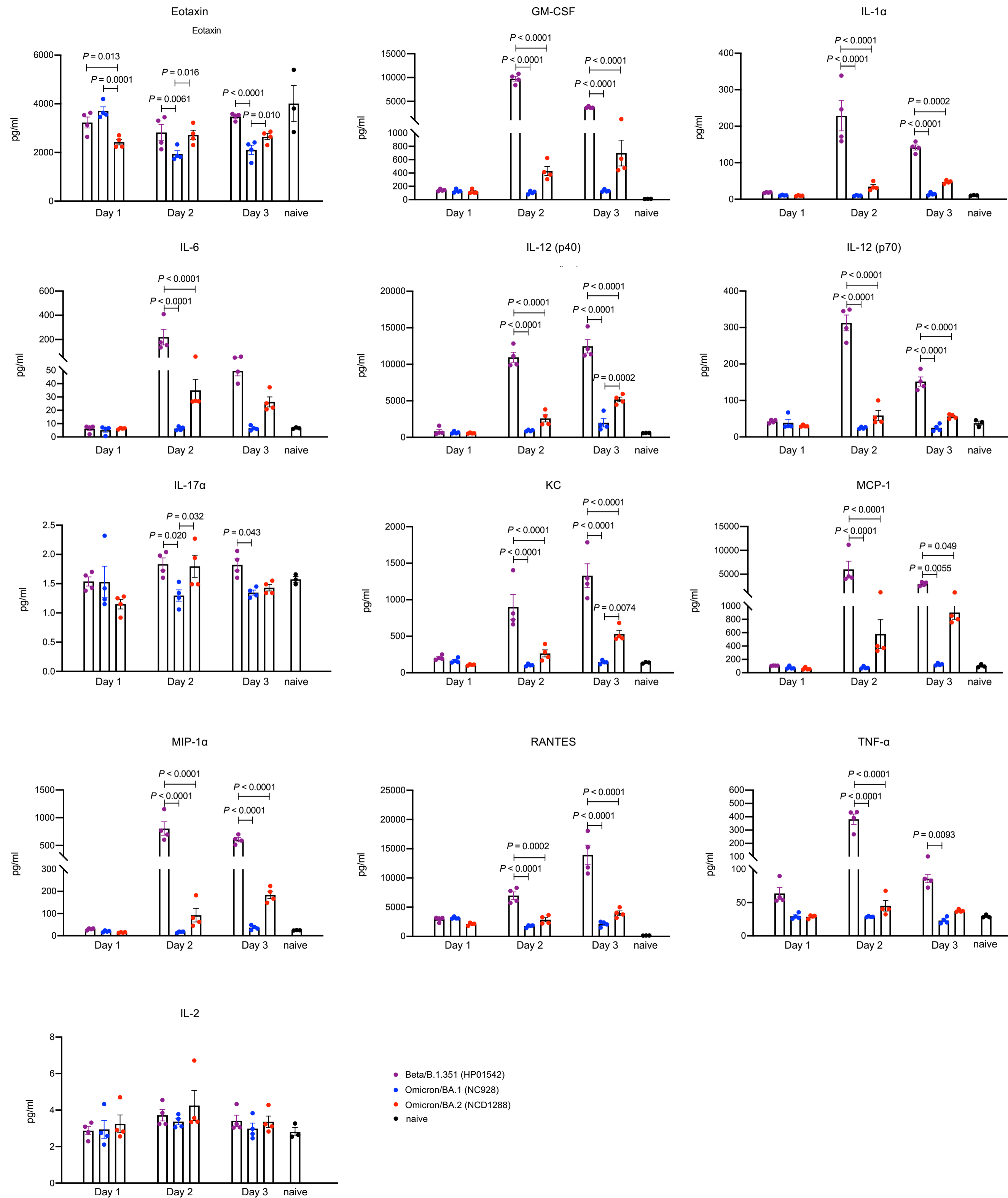

Extended Data Figure 2

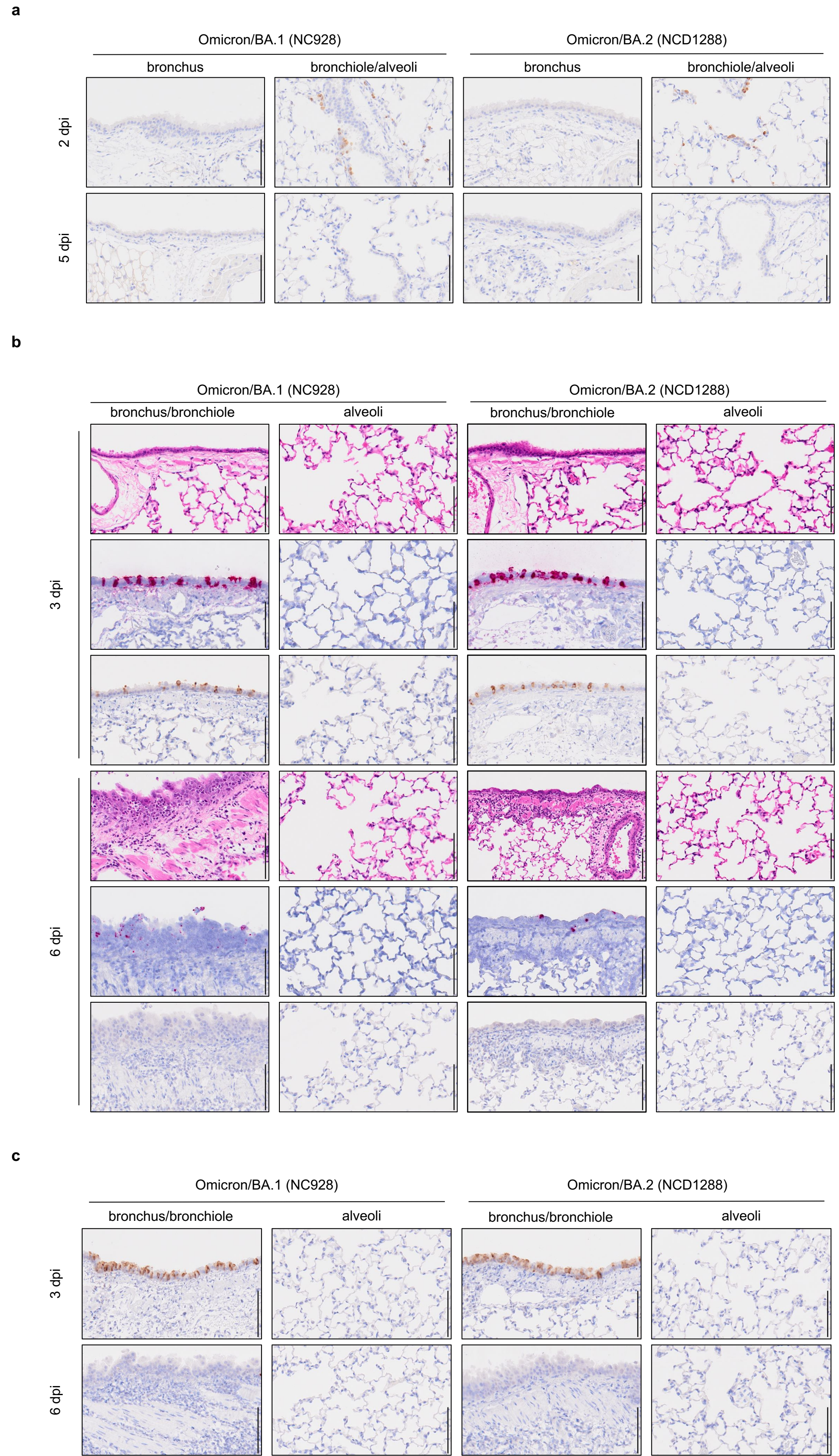

Extended Data Figure 3

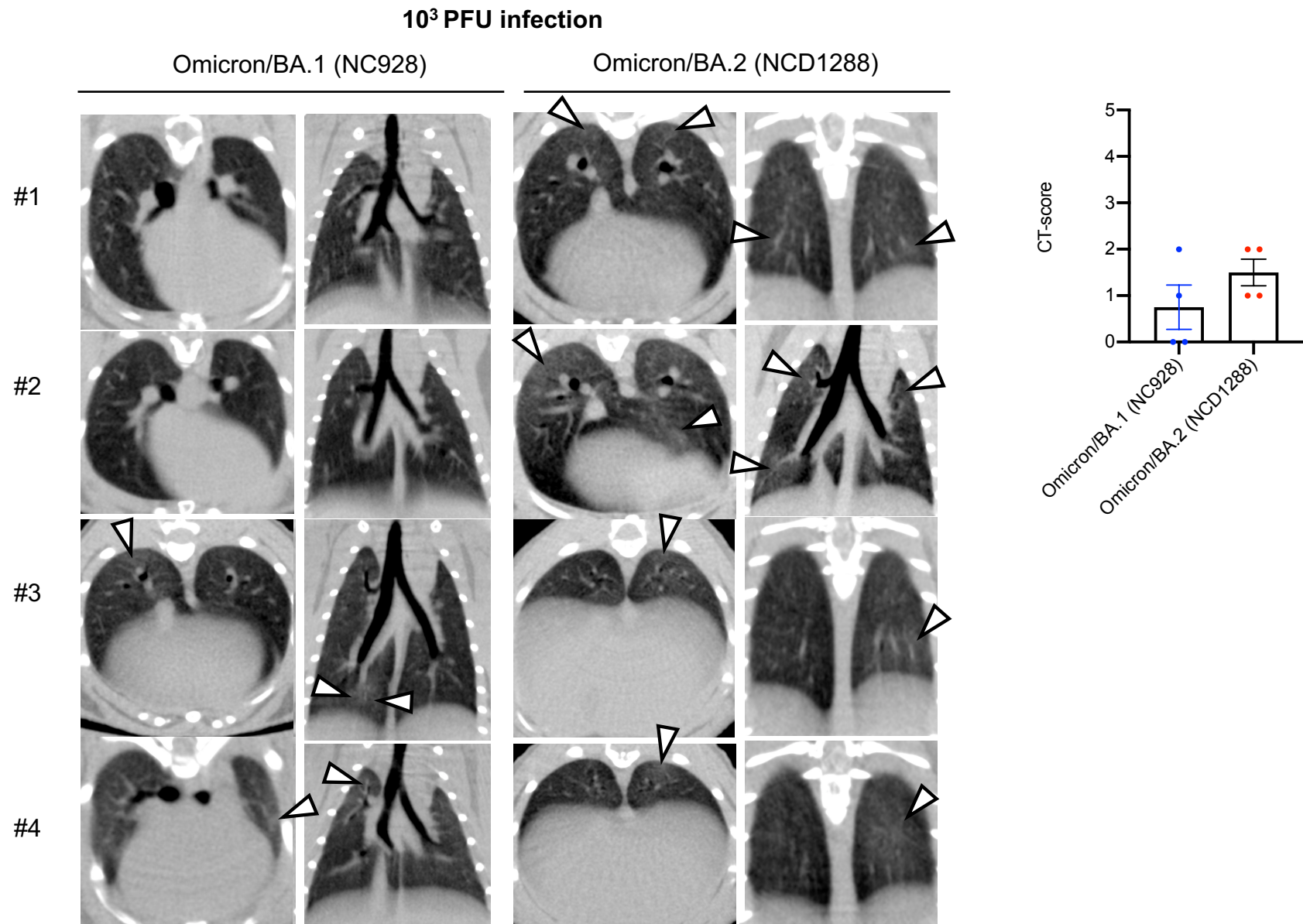

Supplement: Supplement 1 [file 5c8f9a88613e8c6752b6c01e.pdf]
