# Supplementary material for: Characterization and antiviral susceptibility of SARS-CoV-2 Omicron/BA.2
Source: Res Sq. 2022 Feb 24:rs.3.rs-1375091. Preprint. [Version 1] doi: 10.21203/rs.3.rs-1375091/v1 (PMC8887076; doi:10.21203/rs.3.rs-1375091/v1)
Supplement: Supplement 3 [file 93444c445ea1deb8d81190f3.docx]

**Extended Data Figure 1| Host responses in the lungs of mice infected with SARS-CoV-2 Omicron/BA.2.**

Balb/c mice were intranasally infected with Beta/B.1.351 (HP01542), Omicron/BA.1 (NC928), or Omicron/BA.2 (NCD1288). Pro-inflammatory cytokine/chemokine responses in the lungs of the infected mice were assessed at 1, 2, or 3 dpi (infected mice, *n* = 4; naïve mice; *n* =3). Vertical bars show the mean ± s.e.m. Points indicate data from individual mice. Data were analyzed by two-way ANOVA with Tukey's multiple comparisons test.

**Extended Data Figure 2| Pathological findings in the lungs of SARS-CoV-2-infected animals, related to Fig. 1d and Fig. 2f.**

**a,** Immunohistochemistry of the lungs of SARS-CoV-2-infected mice. Three mice per group were inoculated with 10^5^ PFU of Omicron/BA.1 (NC928) or Omicron/BA.2 (NCD1288) and sacrificed at 2 or 5 dpi. Immunohistochemical examinations were performed with a rabbit polyclonal antibody for SARS-CoV nucleocapsid protein, which cross-reacts with SARS-CoV-2 nucleocapsid protein. Representative images of the bronchi and bronchiole/alveoli of mice infected with BA.1 or BA.2 are shown. Scale bars, 100 µm. **b,** Four hamsters per group were inoculated with 10^3^ PFU of Omicron/BA.1 (NC928) or Omicron/BA.2 (NCD1288) and sacrificed at 3 or 6 dpi for histopathological examination. Representative images of the bronchi/bronchioles and alveoli of hamsters infected with BA.1 or BA.2 are shown. Upper panels, hematoxylin and eosin (H&E) staining. Middle panels, *in situ* hybridization targeting the nucleocapsid gene of SARS-CoV-2. Lower panels, immunohistochemistry with a rabbit polyclonal antibody that detects SARS-CoV-2 nucleocapsid protein. Scale bars, 100 µm. **c,** Immunohistochemistry of the lungs of SARS-CoV-2 infected hamsters. Four hamsters per group were inoculated with 10^5^ PFU of Omicron/BA.1 (NC928) or Omicron/BA.2 (NCD1288) and sacrificed at 3 or 6 dpi. Immunohistochemical examination were carried out with a rabbit polyclonal antibody that dtects SARS-CoV-2 nucleocapsid protein. Representative images of the bronchi/bronchioles and alveoli of hamsters infected with BA.1 or BA.2 are shown. Scale bars, 100 µm.

**Extended Data Figure 3| micro-CT images in the lungs of SARS-CoV-2-infected Syrian hamsters, related to Figure 2e.**

Representative micro-CT axial and coronal images of the lungs of four hamsters per group inoculated with 10^3^ PFU of Omicron/BA.1 or 10^3^ PFU of Omicron/BA.2 at 7 dpi. Lung abnormalities included minimal, patchy, ill-defined, peri-bronchial ground glass opacity (white arrowheads), and few, small, focal rounded/nodular regions (black arrows), consistent with minimal pneumonia. Coronal CT images were reformatted to optimize lesion visualization. CT severity scores for hamsters inoculated with 10^3^ PFU of Omicron/BA.1 (*n =* 4) or 10^3^ PFU of Omicron/BA.2 (*n =* 4) were analyzed by using the unpaired student’s t-test. Vertical bars show the mean ± s.e.m. Points indicate data from individual hamsters.
